# Predicting Individualized Joint Kinematics Over Continuous Variations of Walking, Running, and Stair Climbing

**DOI:** 10.1109/OJEMB.2023.3234431

**Published:** 2023-01-05

**Authors:** Emma Reznick, Cara Gonzalez Welker, Robert D. Gregg

**Affiliations:** Department of RoboticsUniversity of Michigan 1259 Ann Arbor MI 48109 USA; Department of Mechanical EngineeringUniversity of Colorado Boulder 1877 Boulder CO 80309 USA

**Keywords:** Biomechanics, gait recognition, assistive devices, assistive robots

## Abstract

*Goal:* Accounting for gait individuality is important to positive outcomes with wearable robots, but manually tuning multi-activity models is time-consuming and not viable in a clinic. Generalizations can possibly be made to predict gait individuality in unobserved conditions. *Methods:* Kinematic individuality—how one person's joint angles differ from the group—is quantified for every subject, joint, ambulation mode (walking, running, stair ascent, and stair descent), and intramodal task (speed, incline) in an open-access dataset with 10 able-bodied subjects. Four N-way ANOVAs test how prediction methods affect the fit to experimental data between and within ambulation modes. We test whether walking individuality (measured at a single speed on level ground) carries across modes, or whether a mode-specific prediction (based on a single task for each mode) is significantly more effective. *Results:* Kinematic individualization improves fit across joint and task if we consider each mode separately. Across all modes, tasks, and joints, modal individualization improved the fit in 81% of trials, improving the fit on average by 4.3${}^{\circ }$ across the gait cycle. This was statistically significant at all joints for walking and running, and half the joints for stair ascent/descent. *Conclusions:* For walking and running, kinematic individuality can be easily generalized within mode, but the trends are mixed on stairs depending on joint.

## I. Introduction

Have you ever been able to recognize a person in the distance just by the way they walk? That is because a person's gait is unique to them and persists over time [1], making gait an excellent biometric for individual recognition [2]. Gait individuality research falls into either gait recognition (e.g., using deep learning) or into the biomechanics realm, where aspects of an individual's gait are used to quantify efficiency, symmetry, and comfort [3], [4], [5]. Consequently, past research has predicted an individual's gait kinematics over level ground by looking at anthropometric parameters, such as age, sex, BMI, and bone geometry [6], [7], [8]. As a person walks on more varied terrain, their gait fluctuates with respect to ground slope, load, and speed [1], [3]. While these factors can be modeled continuously to improve predictions over a wide variety of tasks [9], the largest source of error stems from fitting individual gait [10], [11].

Since gait is so distinct between individuals, it is important to take this individuality into account when modeling gait and designing control algorithms for assistive devices, such as robotic exoskeletons and prosthetic legs. These control systems often have user-specific parameters that must be configured by clinicians [12], [13], harnessing their experience to improve gait symmetry, counteract maladaptive compensations, and assist rehabilitation for populations with impaired gait. As devices get more complex, the control and tuning often requires the technical knowledge of an engineer to translate the clinician's prescription into control parameters [11], [13], [14]. Researchers are working on this problem by building tuning interfaces that allow a clinician or user to directly tune the device without an engineer [15], [16], [17]. The decision-making process of clinicians has also been encoded into auto-tuning algorithms for robotic prosthetic leg controllers [18], [19]. Other human-in-the-loop, auto-tuning algorithms optimize parameters with respect to various outcomes, such as metabolic cost or minimizing error to joint angle trajectories [20], [21], [22], [23], [24], [25]. While these are important steps, these methods generally consider only one ambulation mode (walking) at one speed and incline (together defining the specific *task* within the ambulation mode).

With the capability of assistive devices expanding to more activities, the already complex problem of tuning one ambulation mode (e.g., walking) compounds. Tuning the multi-modal controller of a powered knee-ankle prosthesis for a specific user can take a team of engineers and clinicians as long as 5 hours [14], which falls well outside the length of a typical clinical session. The iterative process of individualizing all possible tasks of daily living would be infeasible for both the prosthetist and user. Additionally, many clinics do not have the equipment to test every task (e.g., variable-incline ramps or stairs). We seek to minimize this burden by making assumptions about gait individuality to minimize the number of specific tasks a clinician must tune. We hope to harness trends in able-bodied ambulation to simplify the individualization process for assistive devices that aim to restore able-bodied gait in impaired populations. This paper investigates what assumptions can be made about individuality across tasks and ambulation modes.

In particular, this study expands upon the assumptions proposed in [11]: in able-bodied individuals, the kinematic individuality seen at level-ground walking (i.e., the difference in joint angle between the individual's kinematics and the population average at each point in the gait cycle) is a good estimate for the individuality across all walking speeds and inclines. We investigate how well this assumption holds across other ambulation modes, or whether similar assumptions can be made about individuality within other ambulation modes. Understanding individuality in the able-bodied population can inform the tuning paradigms of powered prostheses and exoskeletons, which typically use able-bodied reference data in an attempt to restore normative leg biomechanics in populations with impaired gait [26], [27], [28], [29]. Simplifying the tuning process through these assumptions will make these devices more clinically viable by minimizing the time, technical expertise, and testing equipment necessary to configure them [15], [30].

Section II will discuss the multi-activity dataset used to perform the individuality analysis, the analytical procedure, and the statistics used to interpret the results. Section III reports the results of our analysis. Then, in Section IV, we discuss the specific assumptions supported by the data and how each of the investigated sources of variance affect individualization. Finally, Section V gives concluding remarks about how these results can be incorporated into modern tuning paradigms for wearable robots as well as biomechanics research.

## II. Methods

### A. Data Set

The data used for this study was collected for use in assistive device design, and is accessible for download from Figshare [31]. This dataset reports lower-limb kinematics and kinetics of ten able-bodied participants walking at multiple inclines ($\pm 0 {}^{\circ }$, 5${}^{\circ }$, and 10${}^{\circ }$) and speeds (0.8, 1, 1.2 m/s), running at multiple speeds (1.8, 2, 2.2, and 2.4 m/s), and stair ascent/descent with multiple stair inclines (20${}^{\circ }$, 25${}^{\circ }$, 30${}^{\circ }$, and 35${}^{\circ }$). The experimental protocol and subject details can be found in [32]. Though the dataset also reports transitional and non-periodic motions, this paper investigates only steady-state locomotion over multiple ambulation modes (i.e., walking, running, stair ascent, and stair descent) and their task variations (i.e., speed and incline). For each subject in this dataset, each walking and running task contains 30 seconds of steady-state strides, and each periodic stair climbing task contains 5 strides (collating left and right strides assuming symmetry).

### B. Quantifying Individuality

**Figure 1.**
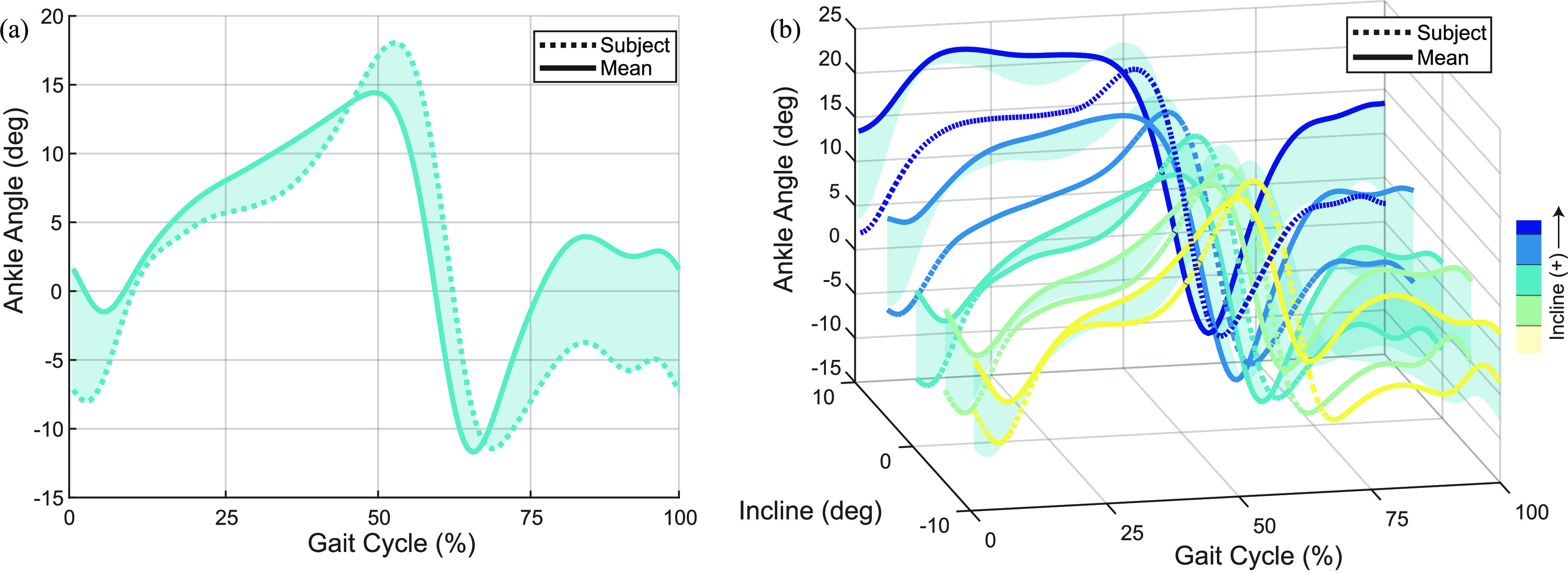
The Individual Kinematic Contribution (IKC) represents the difference between a subject's joint angle kinematics (dashed) and the inter-subject mean (solid) at the same speed and incline. The IKC is calculated at a representative “baseline” task (a; shaded) and then added to other tasks (e.g., different inclines) to predict individuality over the task space (b).

We define an individual's kinematic contribution (IKC) for a given joint at a given task as the difference between the individual's joint kinematics ($\mathbf {d}$) and the leave-one-out average (LOO; $\bar{\mathbf {d}}$) of the other nine subjects (see Fig. 1(a)). In mathematical terms, the IKC is calculated by

**Figure 2.**
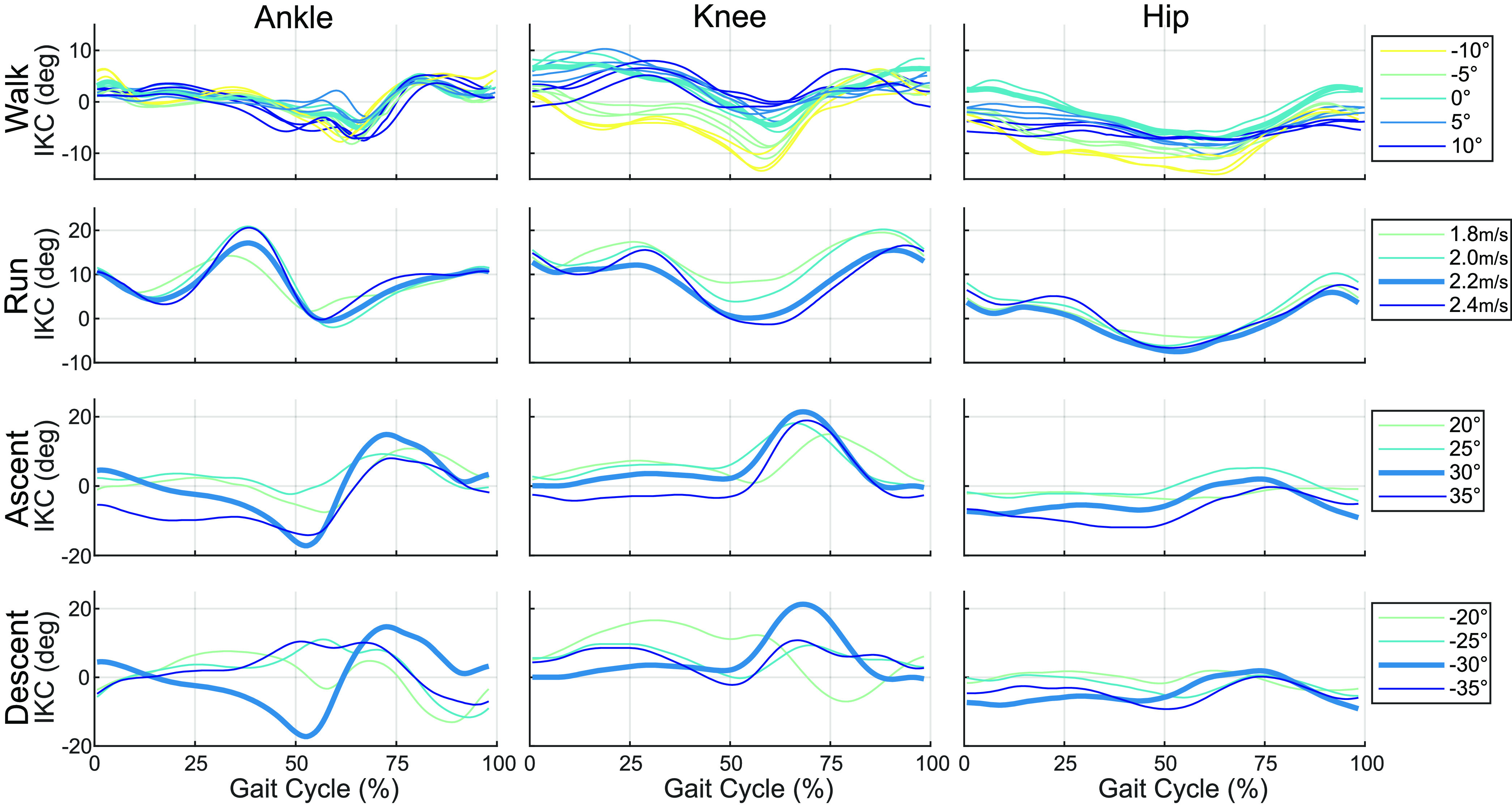
Individual Kinematic Contributions for one subject across all tasks within each joint and ambulation mode. The columns show each of the joints, and the rows correspond to activity (walking, running, stair ascent and descent respectively). The bolded trajectory is the representative task used as the modal baseline. Note the similarities and differences between tasks and modes.

\begin{equation*}
C_{\varphi, \chi, \eta } = \mathbf {d}_{\varphi, \chi, \eta } - \bar{\mathbf {d}}_{\varphi, \chi }, \tag{1}
\end{equation*}

for gait phase $\varphi =1,\ldots,150$, task $\chi =1,\ldots,N$, and subject $\eta = {1, \ldots, 10}$, where the number of tasks $N$ depends on the ambulation mode. The IKC of a representative subject across modes and tasks is shown in Fig. 2.

### C. Individualization of Ambulation Modes

The objective of this study is to individualize multiple ambulation modes using subject-specific data at a minimal number of tasks. Therefore, we calculate individuality for a select few tasks (i.e., baselines) and predictively individualize non-baseline tasks by adding this IKC to the population average (see Fig. 1(b)). We chose the baseline tasks of walking and running over level ground, and stair ascent and descent at an incline representative of a typical stairway. Specifically, we assign the following *modal baseline* tasks: the walking baseline $\chi _{W}$ is 1.0 m/s over level ground, the running baseline $\chi _{R}$ is 2.0 m/s (also over level ground), and the stair ascent and descent baselines, $\chi _{A}$ and $\chi _{D}$, are defined at $\pm 30$${}^{\circ }$, respectively. These tasks are in the middle of each modal task space (i.e., not the fastest, slowest, or most inclined task), and can be easily replicated in a clinic (i.e., parallel bars, hallways, and staircases).

In this study, we predict individuality using two methods. The first method tests and expands the assumption made in [11]: that the *walking baseline* (IKC calculated at $\chi _{W}$) provides a good estimate of individuality across all walking tasks; and extending this assumption to test whether it can be used to predict individuality across other ambulation modes as well. The second method uses *modal baselines* to predict individuality (i.e., walking tasks are estimated by the IKC at $\chi _{W}$ and stair ascent tasks by the IKC at $\chi _{A}$).

### D. Statistics

Throughout this paper, we discuss error using the root mean squared error (RMSE). This metric calculates the deviation of the predicted IKC $C_{\varphi, \chi _{B}, \eta }$ (with respect to baseline $\chi _{B}$) from the subject's observed IKC $C_{\varphi, \chi, \eta }$ by

\begin{equation*}
\text{RMSE}_{\chi, \eta } = \sqrt{\sum\nolimits _{\varphi =1}^{I}(C_{\varphi, \chi _{B}, \eta } - {C}_{\varphi, \chi, \eta })^{2}/I}, \tag{2}
\end{equation*}

for $I = 150$ points in phase. Though we explored IKC fit at specific points along the gait cycle in [11], here we examine the average error across the gait cycle to facilitate comparisons between methods. It should be noted that this single measure cannot differentiate between errors caused by a large, brief residual (e.g., due to a phase shift in swing knee flexion) vs. small persistent differences over the gait cycle. The RMSE is reported in deg, and the results should be interpreted within the context of the respective joint.

As a benchmark, we compare the RMSE from each individualization technique to the RMSE with $C_{\varphi, \chi _{B}, \eta }=0$ in (2), corresponding to the case of no individualization from the population average. Because our input dataset contained a relatively large number of representative walking and running strides collected on a treadmill (about 30 strides over 30 seconds), these models used half of the strides to calculate the baseline IKC and the other half for prediction validation to prevent overfitting. The baseline stair ascent/descent tasks did not have enough strides (5 total periodic strides per task) to split them into separate data for IKC training and validation, so the baseline task was used for training only and omitted from analysis. We define an improvement as a reduction in the individualized RMSE from the non-individualized RMSE, which is quantified by the difference in RMSE (positive values correspond with improvement). We analyze the RMSE between the predicted and observed individuality for each ambulation mode separately with an N-way ANOVA (MATLAB 2021a, Mathworks, Natick, MA) considering the factors of subject$^{1}$, velocity$^{2}$, incline$^{3}$, joint$^{4}$, and individualization method$^{5}$ where applicable. The included factors for each mode are as follows: walking$^{1,2,3,4,5}$, running$^{2,4,5}$, stair ascent$^{1,3,4,5}$, and stair descent$^{1,3,4,5}$. The modal baseline task was removed from the stair models to prevent the omitted task from affecting the accuracy of the ANOVA model. Finally, we use a post-hoc multiple comparison test (Tukey-Kramer) to investigate the effect of individualization methods within specific variable groups.

## III. Results

This section discusses the results from the ANOVA models for each ambulation mode. Specifically, we discuss the group means calculated by the model, the effect of different testing conditions on the resulting RMSE, and significance indicated in post-hoc tests comparing the individualized and non-individualized (average) trajectories to subject-specific experimental kinematics (Table 1).

**TABLE 1:** Post-Hoc Significance Tests Comparing RMSE of Individualized (WB and MB) Vs. Experimental Kinematics With RMSE of Non- Individualized Vs. Experimental Kinematics. Values Below 0.05 (shaded) are Statistically Different, and Values Below $1\mathrm{e}{-4}$ Are Shown as Zero

	Walk	Run		Ascent		Descent	
	MB	WB	MB	WB	MB	WB	MB
Ankle	0.000	1.000	0.002	0.997	0.789	1.000	0.019
Knee	0.000	0.998	0.000	0.999	0.631	1.000	0.172
Hip	0.000	0.000	0.000	0.000	0.000	0.000	0.000

### A. Walk

**Figure 3.**
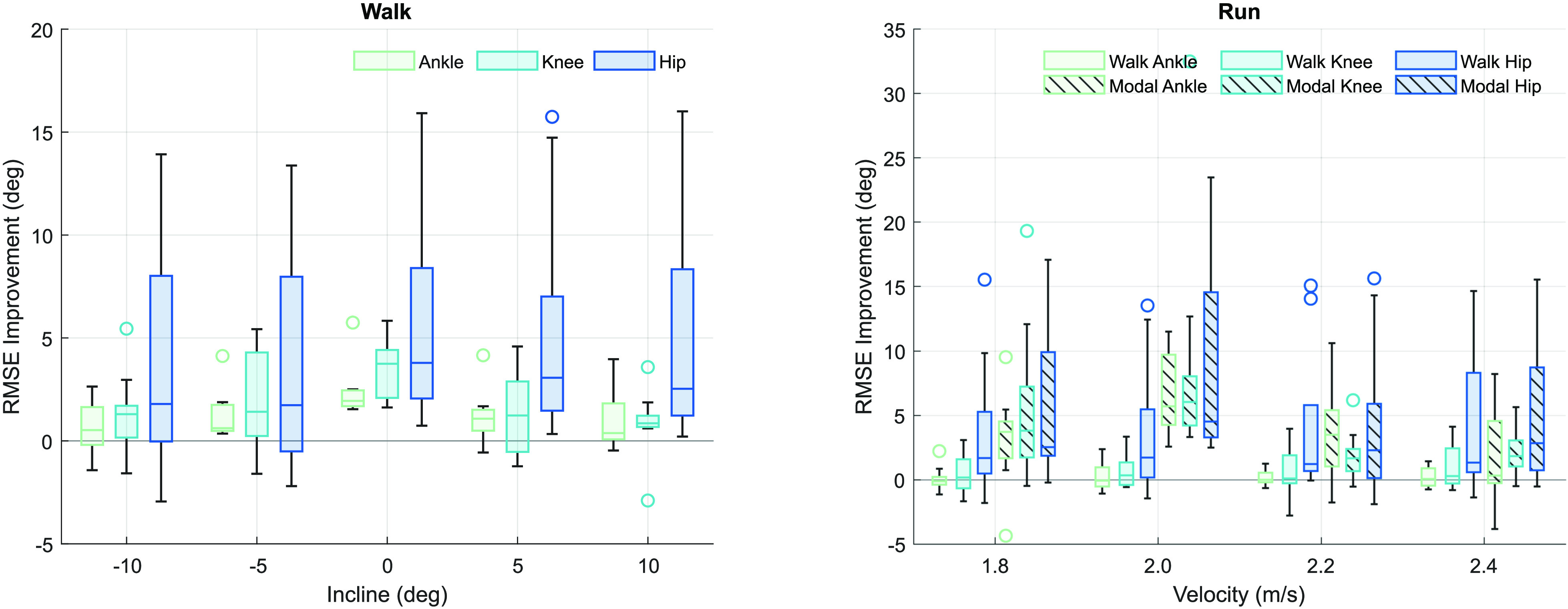
Box plots of RMSE improvement after individualization for walking and running across all joints and subjects. The solid fill indicates walking baseline individualization, and the striped fill represents modal individualization. The boxes show the 25--75% interquartile range, with a line at the median; the whiskers show the non-outlier maximums. We only show RMSE for 1 m/s walking data because velocity had no significant impact on individualization.

In [11], we assumed that the magnitude of the individual kinematic contribution (IKC) at the level ground task was a good estimation of individuality in the dataset presented by Embry et al. [33]. Here, we validate that estimation on a broader, multi-activity dataset [31] to see how far the assumption holds. This validation for walking (seen in Fig. 3, *Walk*) is upheld, showing a significantly improved RMSE at the ankle (1.07${}^{\circ }$, or a 32.7% improvement across all tasks) and the knee (1.53${}^{\circ }$ (29.5%). We see even larger significant improvements at the hip (4.75${}^{\circ }$/63.0%). For this ambulation mode, the walking baseline is equivalent to the modal baseline, so only one set of RMSEs was calculated. The results of the ANOVA suggest that subject, incline, joint, and individualization method all significantly affected the results ($p \ll 0.05$), but velocity had no effect on the resulting RMSE.

### B. Run

For running (and the subsequent stair ambulation modes), two types of individualization were investigated: walking baseline and modal baseline (Fig. 3, *Run*). At the ankle, modal individualization significantly improved the RMSE by 3.75${}^{\circ }$ (59.2% improvement), while using the walking baseline had no significant effect, incidentally improving the fit by 0.18${}^{\circ }$ (2.9%). Similarly at the knee, modal individualization significantly improved the RMSE, while the walking baseline did not; modal individualization improved fit by 4.68${}^{\circ }$ (61.4%), and walking individualization showed a slight improvement of 0.65${}^{\circ }$ (8.5%). At the hip, there was a significant improvement upon individualization of any kind, improving fit for the modal and walking predictions by 5.84${}^{\circ }$ (73.3%) and 4.06${}^{\circ }$ (50.9%), respectively. The ANOVA showed that every factor (velocity, joint, and ambulation mode) was significant.

### C. Stair Ascent

**Figure 4.**
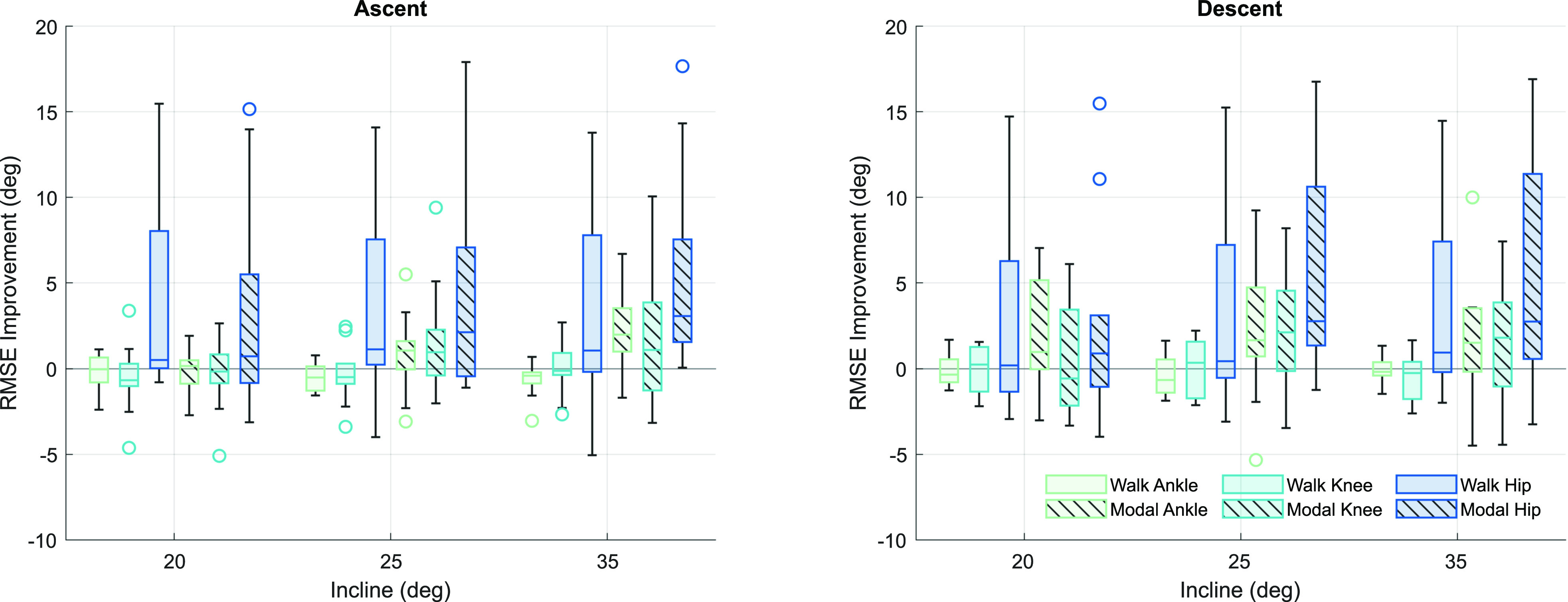
Box plots of RMSE improvement after individualization for stair ascent and descent at the ankle, knee, and hip across all subjects. The solid fill indicates walking baseline individualization, and the striped fill represents modal individualization. The baseline task used for IKC calculation was omitted from prediction (see Section II-D).

For this analysis, we separated the data related to stair ascent and descent because the kinematic individuality presented itself in different parts of the gait cycle. For stair ascent, we found no significant individual kinematic trends at the ankle or knee (Fig. 4, *Ascent*; Table 1). Despite this, modal individualization tended to slightly improve the RMSE across all tasks: 0.90${}^{\circ }$ (19.7%) and 1.04${}^{\circ }$ (16.8%) for the ankle and knee, respectively. Walking individualization slightly worsened the RMSE, an average decrease of 0.45${}^{\circ }$ (9.8%) and 0.31${}^{\circ }$ (5.0%) for the ankle and knee, respectively. The hip had significant improvements with both methods: walking individualization improved RMSE by 3.73${}^{\circ }$ (42.9%) and modal individualization by 4.57${}^{\circ }$ (52.6%). Despite the post-hoc tests showing no specific significance (Table 1), stair inclination, individualization type, subject, and joint significantly affected the results.

### D. Stair Descent

Stair descent (Fig. 4, *Descent*) had no significant improvements using the walking baseline at the ankle or knee, showing small reductions in RMSE of 0.19${}^{\circ }$ (3.2%) and 0.10${}^{\circ }$ (1.5%), respectively. Hip fit improved, similar to stair ascent, by 3${}^{\circ }$ across all subjects (or 40.7%). Interestingly, modal individualization improved RMSE of the ankle for stair descent more than observed with ascent, significantly decreasing the RMSE by 1.94${}^{\circ }$ (31.9%). The knee also improved fit by 1.50${}^{\circ }$ (25.4%), though not significantly. The modal individualization significantly improved the hip by 4.28${}^{\circ }$ (58.2%). In this ambulation mode, the ANOVA model showed subject, joint, and individualization type had a significant effect on RMSE, but stair incline did not.

## IV. Discussion

In this paper, we sought to predict lower-limb kinematic individuality for a variety of different activities with a minimum number of observed, individualized tasks. We compared the predictions of two individualization methods across different modes (walking, running, stair ascent and descent) and tasks (e.g., speed, incline). We found that walking individuality did not statistically improve fit across all modes (despite improving 72% of trials by 2.2${}^{\circ }$ on average), but using one baseline task per mode improved kinematic predictions for walking and running. These findings uphold assumptions for walking made in [11]. The improvements of stair climbing were mixed and improved the hip more than other joints.

Overall, an individual's gait varies consistently, and how they differ from average holds within modes but does not extend across them. In walking and running, inter-task variability within modes is low (Fig. 2), and therefore we see significant decreases in RMSE after modal individualization. Modal individualization improved the fit for 84% of walking trials by 3.1${}^{\circ }$ on average, and improved 90% of running trials by 5.6${}^{\circ }$. Fig. 3 also demonstrates that in trials where individualization decreased fit, the effect was small: 1.0${}^{\circ }$ for walking trials and 1.5${}^{\circ }$ for running.

Though this method worked well for walking and running, it had less success with stair climbing, resulting in statistically significant improvements in individuality primarily at the hip joint. Although the modal stair individualization did not statistically improve all joints, the overall results still demonstrate improvements compared to the non-individualized benchmark (Fig. 4). Modal individualization improved 68% of ascent trials by 3.9${}^{\circ }$ and 71% of descent trials by 4.4${}^{\circ }$. In addition, investigation of Fig. 4 shows that the modal baseline fit is better at higher inclines, but does not match as well at lower inclines. This is corroborated at the trajectory level, where some subjects show different locomotive strategies for high and low incline stairs. Further, these locomotive strategies can functionally alter key kinematic landmarks (i.e., phase shifts), which is not accounted for by this individualization method and is heavily penalized by the RMSE metric.

In this study, kinematic changes associated with velocity and incline, like in [6], [7], are largely accounted for through the IKC calculations. We see that predictions near the baseline task are often best, and the tasks further in the task space show individuality that as well predicted. The predicted level-ground walking IKC seems to better fit inclined walking than declined. Running has similar trends, with lower velocity trials being better fit than higher velocity, especially at the ankle. Interestingly, different gait strategies are used with different stair heights, and the walking baseline is a comparable estimation of low incline stairs;trends in Fig. 4, *Ascent*, show that walking IKC is a better prediction of low-incline trends, while higher inclines are better fit by modal individualization.

Across all ambulation modes, post-hoc tests indicated that modal individualization improved the fit for 9 of the 10 subjects, while one subject only improved fit for non-stair modes. Significance in RMSE improvement between modes is dependent on subject, and inter-task variability is a large factor in the success of kinematic individualization. Subject-specific improvements were dependent on the inter-task variance, but most showed a 1-5${}^{\circ }$ decrease in error after individualization (with some reaching as high as 15${}^{\circ }$). Interestingly, both stair ascent and descent showed subject-wise improvements, but the improvements were consistently more pronounced in descent.

If we investigate variance by joint, it is clear that individualization of the hip is important, accounting for a large portion of gait individuality [34], and showing significant improvements after individualization for every method and ambulation mode. The motion of the hip is largely sinusoidal, and individuality presents as both amplitude and linear shifts in position. The linear shifts in hip angle can stem from placement of the pelvic markers during motion capture, but changes in amplitude and shifts in phase are highly individual. In contrast, the knee showed the highest residuals after individualization, and we believe this stems from the larger range of motion of this joint. The ankle is interesting because the largest areas of individuality stem from magnitude changes corresponding to changes in toe-off phase. We investigated methods to isolate the effect of stance percentage and magnitude individuality, but phase individualization did not improve the current magnitude individualization. This is an area for further study.

While the results are promising, there are limitations to the study. Firstly, this study assumes that the able-bodied subjects in the dataset have perfectly symmetric gait. This is not always the case, leading to a poorer fit on both sides because the IKC is an average of the two legs. Secondly, we choose slightly different methods in our IKC calculation depending on task because of dataset limitations. To prevent dropping rank in the walking and running ANOVA models, we use half of the strides for IKC calculation and the other half for experimental validation. Because there are fewer stairs strides, we use all available strides from the baseline task to calculate the IKC and do not predict this task. Additionally, this study focuses only on able-bodied, stead-state gait, and does not touch on impaired or non-steady motion. Lastly, we acknowledge that the hip significantly improves the results for the generalized analyses across joints. We have separated results whenever possible, so the reader can draw their own conclusions.

The observations in this paper provide a framework for individualizing across a multi-activity task space. Currently, the paradigm for powered prostheses and exoskeletons is individually tuning each joint, task, and ambulation mode. The method of kinematic individualization tested in this study provides a framework for generalizing across tasks within some ambulation modes to minimize the time spent tuning. Future studies will investigate whether the trends also extend to kinetics. Individualization of devices is imperative [13], [14], [15], [18], and mirroring a clinical environment (i.e., minimal equipment and instrumentation) can reduce manual tuning time and increase the clinical viability. Further work will investigate trends in non-steady behavior, and create tuning interfaces that implement these kinematic trends.

## V. Conclusion

This study used a comprehensive dataset to generate hypotheses that can be translated into the clinical field to make powered assistive devices more clinically viable. We found that individualization based on one task per ambulation mode is feasible for steady-state walking and running, but statistical results for stair ascent and descent were mixed. These observations can be integrated into tuning paradigms to decrease the clinical dependence on engineers, and lessen the barriers for adoption of powered assistive devices.
